# Atom-optically synthetic gauge fields for a noninteracting Bose gas

**DOI:** 10.1038/s41377-021-00702-7

**Published:** 2022-01-07

**Authors:** Yuqing Li, Jiahui Zhang, Yunfei Wang, Huiying Du, Jizhou Wu, Wenliang Liu, Feng Mei, Jie Ma, Liantuan Xiao, Suotang Jia

**Affiliations:** 1 State Key Laboratory of Quantum Optics and Quantum Optics Devices, Institute of Laser Spectroscopy, Shanxi University, Taiyuan, 030006 China; 2 Collaborative Innovation Center of Extreme Optics, Shanxi University, Taiyuan, Shanxi 030006 China

**Keywords:** Atom optics, Nonlinear optics

## Abstract

Synthetic gauge fields in synthetic dimensions are now of great interest. This concept provides a convenient manner for exploring topological phases of matter. Here, we report on the first experimental realization of an atom-optically synthetic gauge field based on the synthetic momentum-state lattice of a Bose gas of ^133^Cs atoms, where magnetically controlled Feshbach resonance is used to tune the interacting lattice into noninteracting regime. Specifically, we engineer a noninteracting one-dimensional lattice into a two-leg ladder with tunable synthetic gauge fields. We observe the flux-dependent populations of atoms and measure the gauge field-induced chiral currents in the two legs. We also show that an inhomogeneous gauge field could control the atomic transport in the ladder. Our results lay the groundwork for using a clean noninteracting synthetic momentum-state lattice to study the gauge field-induced topological physics.

## Abstract (web summary)

A two-leg ladder with tunable gauge fields is achieved by one-dimensional synthetic momentum-state lattice of a noninteracting Bose gas.

## Graphical abstract

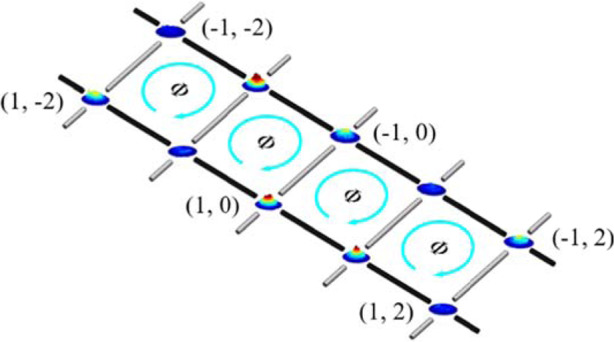

## Introduction

Gauge fields describe the basic interactions between charged particles and are responsible for various emergent topological phases of matter^1,2^. This concept recently has been further generalized to charge neutral systems^3^. Via engineering an analog Hamiltonian that governs the effective dynamics of neutral particles subject to magnetic fields, synthetic gauge fields have been produced in various artificial systems^4–12^. In particular, ultracold atoms have been widely demonstrated as a well-controlled platform for implementing synthetic gauge fields^13–19^, leading to the observation of various topological phases of matter^20–28^. An important development in this regard is implementing synthetic gauge fields in synthetic lattices formed by using the internal states of atoms as lattice spatial dimensions^29–36^. This approach opens a route for exploring high-dimensional topological physics with low-dimensional ultracold atomic systems.

Very recently, an alternative synthetic lattice technique has been proposed by exploiting the momentum states of ultracold atoms as lattice spatial dimensions^37–44^. The hoppings between lattice sites are implemented by driving a series of Bragg transitions to couple discrete momentum states. The key advantage in such synthetic lattice is that all parameters in the realized model can be flexibly tailored by tuning the multiple frequency components of Bragg lasers, including the hopping rates, phases and on-site energies. Benefiting from this feature, multifarious physical models have been experimentally realized in one-dimensional (1D) synthetic momentum-state lattices^38–40^. Moreover, a two-leg momentum-state ladder has been also experimentally formed through two sets of Bragg laser beams, where the chiral currents and atomic reflections induced by synthetic gauge fields were observed^42^. However, all the previously implemented momentum-state lattices are based on the Bose gas of ^87^Rb atoms and with untunable interaction^45^. In particular, for the interaction energy that is comparable to the tunneling strength between the synthetic lattice sites, the interaction-induced nonlinearity would affect the noninteracting single-particle physics^46^.

Here, we experimentally realize a momentum-state lattice based on a Bose gas of ^133^Cs atoms. Compared with ^87^Rb atoms, ^133^Cs atoms with tunable *s*-wave scattering length allow us to obtain a noninteracting lattice for the immaculate investigation of chiral behavior induced by the synthetic gauge fields^45,47^. We implement gauge fields in a two-leg momentum-state ladder that are generated through one set of Bragg laser beams. The gauge fields are synthesized by locally tuning the intra-leg hopping phase. Through measurements of atomic populations in momentum-state sites varying with tunable gauge fields, we clearly observe the chiral behavior and also the variation of chiral atomic current with the inter-leg coupling. Finally, we study the atomic transport in a ladder subject to an inhomogeneous artificial magnetic field. Our experimental results show good agreement with theoretical simulations.

## Results

**Fig. 1:**
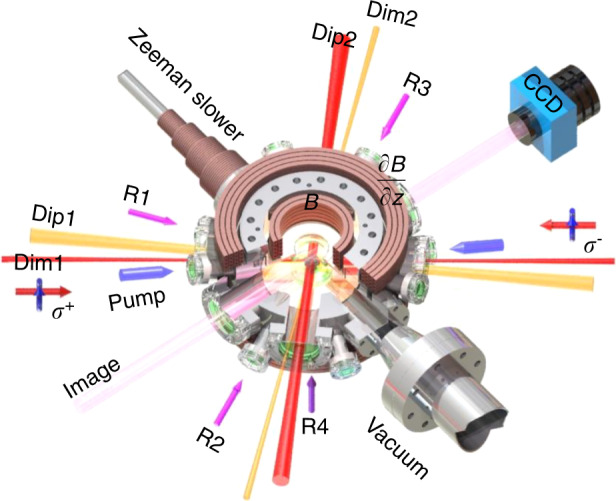
Experimental setup. Four Raman lasers (R1-R4) are used for 3D degenerated Raman sideband cooling, in which the blue detuned pump lasers (pump) are used to polarize the atoms. The crossed dimple trap (Dim1 and Dim2) is overlapped with a magnetically levitated dipole trap (Dip1 and Dip2) for forming a combined optical trap. The hybrid evaporative cooling is implemented by reducing the magnetic field gradient *∂B/∂z* and lowering the powers of the optical trap lasers. A ^133^Cs BEC is finally confined in a quasi-1D optical trap composed of the lasers Dim1 and Dip2. The uniform magnetic field *B* can be tuned to change the atomic interaction. The momentum-state lattice is formed by reflecting the dimple trap laser Dim1 to construct a series of Bragg transitions between discrete momentum states

Three-dimensional (3D) degenerated Raman sideband cooling (DRSC) is first used to prepare cold ^133^Cs atoms in the hyperfine state *F* = 3*, m*_*F*_ = 3^48,49^. Then the hybrid evaporative cooling is implemented for the atoms in a combined optical trap, which is formed by overlapping the crossed dimple trap with the magnetically levitated dipole trap^50^, as shown in Fig. 1. The Bose–Einstein condensate (BEC) is produced in a quasi-1D trap at the scattering length of *a* = 210 *a*_0_ (with *a*_0_ Bohr radius), where the three-body loss is minimized^51–53^. The quasi-1D geometry is provided by one dimple trap laser (Dim1, 1*/e*^2^ radius of ∼58 *µ*m) and another dipole trap laser (Dip2, 1*/e*^2^ radius of ∼300 *µ*m), and this leads to the almost free propagation along the axial direction and the strong radial confinement.

The interaction in ^133^Cs BEC can be widely tuned through a broad magnetic Feshbach resonance centered at −11.7 G^45,47,52^. This makes it possible to obtain a noninteracting synthetic lattice model for accurately studying various noninteracting single-particle physics. To avoid the instability of BEC at the zero scattering length^45,54,55^, the atomic scattering length is linearly ramped to *a* = 3 *a*_0_ in 500 ms. Our experiment starts with a noninteracting BEC of ∼5 × 10^4 133^Cs atoms. The 1D momentum-state lattice is constructed by reflecting the remaining dimple trap laser (Dim1) to drive a series of Bragg transitions between discrete atomic momentum states |*n* > with momenta $p = 2n\hbar k$ (*k* = 2π/*λ* and *ħ* is the reduced Planck’s constant)^37,56^. Two acoustic optical modulators (AOMs) are used in the reflected laser beam to generate the multi-frequency components with *ω*–Δ*ω*_*n*_, while the incident laser has a single frequency *ω*. The Bragg resonance frequency is $\Delta \omega _n = \omega _n^{{\rm{res}}} = (2n + 1)4E_R/\hbar$ for two adjacent momentum states, where *E*_*R*_ = *ħ*^2^*k*^2^/2*m* is the one-photon recoil energy and *m* is the mass of ^133^Cs atom.

**Fig. 2:**
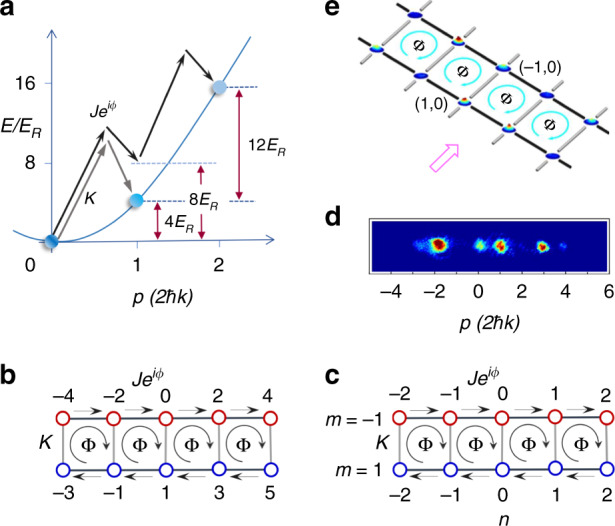
Synthetic noninteracting two-leg ladder with tunable gauge fields. **a** Illustration of atomic energy diagram and the controllable couplings between discrete momentum states. Bragg transitions with two- and four-photon processes are used to couple the nearest-neighbor (NN) and next-nearest-neighbor (NNN) momentum states, respectively. **b** A two-leg ladder geometry with 2 × 5 sites is obtained by selectively controlling the NN and NNN couplings in a noninteracting 1D momentum-state lattice. $J{\rm{e}}^{{\rm{i}}\phi }$ and *K* represent the intra- and inter-leg hopping rates. Φ = 2*ϕ*. is synthetic magnetic flux and can be directly tuned by varying the tunneling phases in the legs. **c** The lattice sites in (b) are re-encoded to give the coordinates (*m, n*) in a two-leg ladder. **d** The absorption image after 18 ms time of flight shows the density distribution of atoms with different momenta in one dimension. **e** Population of atoms in the synthetic two-leg ladder can be extracted through the connection of the different momentum states to the re-encoded lattice sites (*m, n*)

The schematic for forming synthetic gauge fields in a two-leg ladder is presented in Fig. 2. As shown in Fig. 2a, in addition to the standard two-photon Bragg transition used for coupling the nearest-neighbor (NN) momentum states, a four-photon Bragg transition is further introduced for coupling the next-nearest-neighbor (NNN) momentum states by using two pairs of lasers with the same frequency difference $\Delta \omega = \frac{1}{2}[(2n - 1) + (2n + 1)]4E_R/\hbar$. As depicted in Fig. 2b, by designing the specified NNN and NN Bragg couplings in the momentum-state lattice, a two-leg ladder can be directly constructed from a single 1D momentum-state lattice. Note that the design of NNN couplings is also important for realizing a Zigzag momentum-state lattice^41^. The effective tunneling phase *ϕ* mimicking the gauge flux in the ladder is implemented and tuned by controlling the laser phases in the four-photon Bragg processes. In Fig. 2c, a 2 × 5 site ladder is obtained by re-encoding the coordinates of the lattice sites shown in Fig. 2b, with *m* and *n* representing the synthetic directions along the rung and leg, respectively. In this ladder system, all hoppings along the legs and rungs can be addressed individually. Moreover, the use of noninteracting BEC avoids the interaction-induced on-site energy shift in the synthetic ladder, and eliminates the nonlinear effect on the single-particle lattice model Hamiltonian^46,57,58^. Figure 2d presents the absorption image after 18 ms time of flight (TOF), in which the atomic populations in the different momentum states can be extracted directly. The TOF image in Fig. 2d is arranged to show the density distribution of atoms in the two-leg ladder in Fig. 2e, according to the connection of the atomic momenta to the synthetic lattice sites shown in Fig. 2b.

Similar to the motion of charged particles in the periodic lattice subject to a real magnetic field, the Hamiltonian describing the dynamics of ultracold neutral atoms in the synthetic two-leg ladder subject to synthetic gauge fields is given by

1 $$\hat H = J\mathop {\sum}\limits_{m,n} {{\rm{e}}^{{\rm{i}}\phi _{m,n}}\hat c_{m,n + 1}^{\dagger} \hat c_{m,n}} + K\mathop {\sum}\limits_n {\hat c_{1,n}^{\dagger} \hat c_{ - 1,n}} + {\rm{h.c.}}$$

where $\hat c_{m,n}$ and $\hat c_{m,n}^{\dagger}$ are the annihilation and creation operators at the site (*m*, *n*), *J* is the intra-leg tunneling energy between two adjacent sites, *K* is the inter-leg coupling strength along the vertical rung, and the effective tunneling phases along the legs are given by $\phi _{m,n} = - m\phi$ with $m \in \left\{ { - 1,_{}1} \right\}$. Since the atomic scattering length is tuned to near zero, the interactions can be ignored and the above tight-binding Hamiltonian is intact. The tunneling phase *ϕ*_*m,n*_ can be locally engineered through the programmable multi-frequency rf signal used for driving the AOM. The sum of the tunneling phases around a four-site plaquette gives the synthetic gauge flux Φ. The synthetic magnetic field *B* can be obtained by $\Phi _{AB}/2{\pi} = \ell ^2B/\Phi _0$, where the Φ_*AB*_ is the Aharonov–Bohm phase acquired by a particle with the charge *q*, $\ell$ is the lattice constant and Φ_0_ = 2π*ħ*/*q* is the magnetic flux quantum^18,31^.

**Fig. 3:**
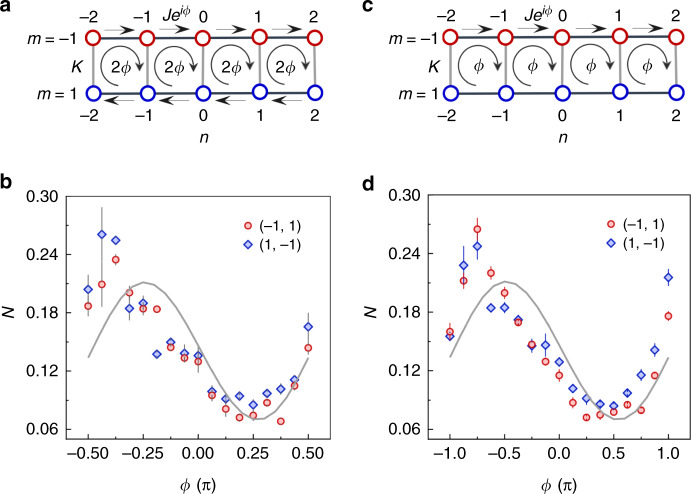
Dependence of atomic population on the gauge phase in a two-leg ladder. **a** Schematic of a two-leg ladder with a gauge flux Φ = 2*ϕ* in each plaquette. **b** The initial sate is prepared in an equal superposition of two momentum-state sites (−1, 0) and (1, 0). The populations on the sites (−1, 1) and (1, −1) are measured after a duration of *t* = 160 *µ*s as a function of gauge phase *ϕ*. **c** shows the synthetic gauge flux $\Phi = \phi$ when the tunneling phases in the leg *m* = 1 are turned off. **d** The populations on the sites (−1, 1) and (1, −1) corresponding to the case in (**c**) after the same duration *t*. Solid lines in (**b**), (**d**) represent the results from the numeric simulation of noninteracting tight-binding models. The intra- and inter-leg coupling energies are set as *J/ћ =* 2π × 500 Hz and *K/ћ =* 2π × 500 Hz, respectively

We firstly apply a homogeneous synthetic gauge field in the two-leg ladder and demonstrate the presence of chiral behavior in terms of the population of atoms in two lattice sites with inverse symmetry. Compared to the only tunability in one leg in ref. ^42^, our system has controllable inter and intra-leg couplings, and the synthetic gauge field can be implemented by tuning the tunneling phases in both up and down legs. Through a π/2-pulse the noninteracting BEC is prepared into an equal superposition of two momentum states *p* = 0 and *p* = 2*ħk* corresponding to the sites (−1, 0) and (1, 0). As described in Fig. 3a, the flux is applied by introducing a gauge phase *ϕ* in both the *m* = −1 and *m* = 1 legs, producing a homogeneous gauge flux Φ = 2*ϕ* in each plaquette. After the preparation of the initial superposition state, the system is quenched into the ladder system with synthetic gauge fields. Figure 3b shows the response of the atomic populations in the sites (−1, 1) and (1, −1) to the applied gauge flux after a duration of *t* = 160 *µ*s. We find that the atomic populations have a periodic oscillation with the gauge phase *ϕ*, reflecting that a homogeneous gauge field could induce atomic current. In terms of the chirality, the atomic population in the site (−1, 1) displays the same dependence on the gauge flux with that in the site (1, −1).

We also study the response of the atomic population to another homogeneous gauge field, where the gauge phase in the *m* = 1 leg is turned off, as shown in Fig. 3c. The resulting gauge flux is Φ = *ϕ* in each plaquette in this case. Figure 3d shows that the period of the variation of atomic population with the gauge phase *ϕ* is twice as long as the one in Fig. 3b, in accordance with the effective gauge field theory. The experimental data are in reasonable agreement with the theory curve, which are obtained by magnifying the numerical simulation with a factor of 1.5. The main deviation is attributed to both the decoherence of atomic BEC and the NNN couplings induced off-resonant Bragg transitions between the lattice sites in each four-site plaquette.

**Fig. 4:**
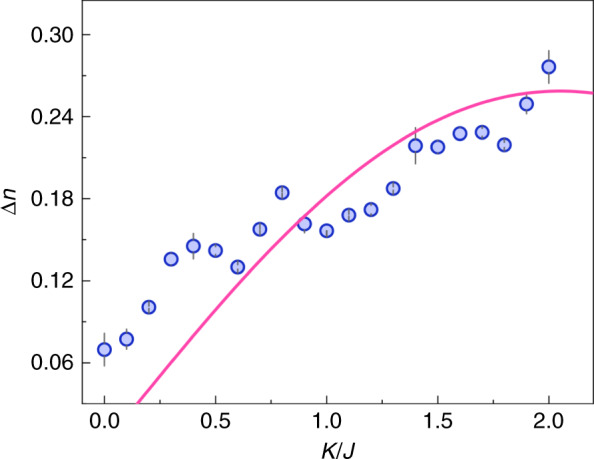
Dependence of chiral atomic current on the inter-leg coupling. The population imbalance ∆*n*, characterizing the chiral atomic current in the ladder, as a function of the inter-leg coupling *K* after an evolution time of 160 *µ*s. The intra-leg tunneling energy is *J/ћ* = 2π × 500 Hz and the gauge flux is Φ = π/4. The solid line represents the theoretical result from the numerical simulation of a noninteracting tight-binding model

We further go to study the effect of the inter-leg coupling along the rung on the chiral feature of atomic current. In the momentum-state lattice, the chiral atomic current in the two-leg ladder can be characterized by the population imbalance in the two legs and is defined as

2 $$\Delta n = \left\langle n \right\rangle _{ - 1} - \left\langle n \right\rangle _1$$

where $\left\langle n \right\rangle _m = \mathop {\sum}\nolimits_n {P_{m,n}} \times n$ characterizes the strength of the chiral motion of the particles along the leg *m*^32,42^. Figure 4 shows the measurement of the chiral current ∆*n* as a function of the inter-leg coupling *K* after an evolution time of *t* = 160 *µ*s for the gauge flux Φ = π/4. As expected, we observe a vanishing chirality when the intra-leg couplings are turned off, even in the presence of a gauge flux; When the intra-leg couplings turn on and increase, the chiral feature becomes stronger; The numeric calculation indicates that ∆*n* will decrease with increasing *K* for *K* > 2*j*, and the chirality is suppressed when $K > > J$. To practically satisfy the condition $K(J) < < 8E_R$ for the momentum-state lattice, the tunneling energy is kept below 0.8 *E*_*R*_ in our measurement. The solid line is the corresponding theoretical prediction, where the numeric simulation result is scaled by a factor of ∼0.84.

**Fig. 5:**
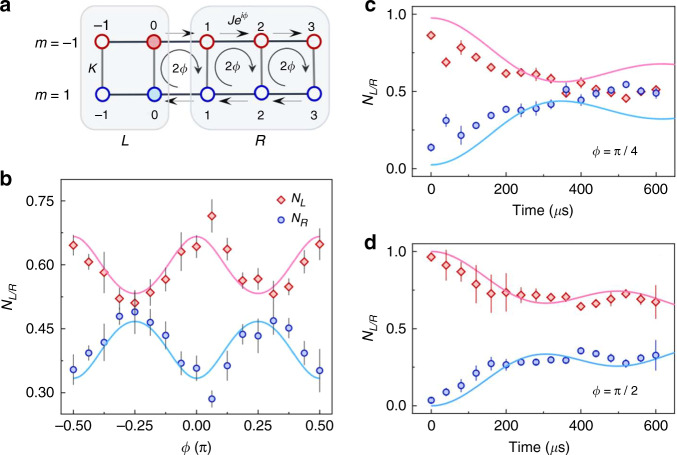
Atomic transport in an inhomogeneous artificial magnetic field. **a** Schematic of the two-leg ladder with an inhomogeneous artificial magnetic field produced by applying a step-changing gauge flux. The ladder is divided into two parts, in which the leftmost plaquette (rightmost two plaquettes) with zero (nonzero) flux is referred as the left (right) cell. **b** The population of atoms in the left and right cells (*N*_*L*_ and *N*_*R*_) as a function of the gauge phase *ϕ* after an evolution time of *t* = 320 *µ*s. Time evolution of *N*_*L*_ and *N*_*R*_ for (**c**) *ϕ* = π/4 and (**d**) *ϕ* = π/2. Solid lines are theoretical curves from numerical simulation of noninteracting tight-binding models

Finally, we study the transport of atoms in a ladder subject to an inhomogeneous artificial gauge that is generated by using a step-like gauge flux. In comparison with homogeneous artificial gauge fields, the atomic transport has a substantially different behavior under inhomogeneous gauge fields. As shown in Fig. 5a, the gauge flux distribution in the ladder can be designed by locally controlling the tunneling phases in the up and down legs. In our experiment, the gauge flux in the leftmost plaquette is designed as Φ_L_ = 0, and the gauge fluxes in the rest of plaquettes are Φ_*R*_ = 2*ϕ*. In this way, the two-leg ladder is divided into two cells as depicted in Fig. 5a. Likewise, we prepare the atoms into an equal superposition of two sites (−1, 0) and (1, 0), and quench the system into a ladder with inhomogeneous gauge fields. The atomic transport is measured by monitoring the time evolution of atomic population in the left and right cells (*N*_*L*_ and *N*_*R*_). Figure 5b shows the dependence of *N*_*L/R*_ on the *ϕ* after an evolution time of *t* = 320 *µ*s with all atoms initialized in the left cell. When the gauge phase *ϕ* is tuned from −π/2 to π/2, the population of atoms in the left and right cells have the oppositely periodic oscillation behaviors. We find that *N*_*L*_ approaches the maximal value when the gauge flux is tuned to Φ = 2*ϕ* = 0, ±π. The maximal atomic population for *N*_*L*_ is less than 0.7 due to the bidirectional transport. When the gauge flux is tuned to Φ = 2*ϕ* = ±π/2, a half of atoms in the left cell transport to the right cell, and *N*_*R*_ arrives the maximum value around 0.5. The experimental results are in good agreement with the theoretical calculations based on the tight-binding Hamiltonian in Eq. 1.

The atomic transport from the left cell to the right one is further exhibited in detail by observing the time evolution of *N*_*L*_ and *N*_*R*_ for two specific gauge phases *ϕ* = π/4 (Fig. 5c) and *ϕ* = π/2 (Fig. 5d). The observed atomic transport is in good agreement with the theoretical prediction. The increase of *N*_*R*_ (decrease of *N*_*L*_) with time indicates the transmission of atoms from the left cell to the right one through the central synthetic boundary. The transmission is inhibited after *t* ∼ 300 *µ*s and the atomic distributions in the two cells reach an equilibrium. Moreover, compared to the case for *ϕ* = π/2, a significant transmission occurs for *ϕ* = π/4. Therefore, our experiment clearly demonstrates that an inhomogeneous gauge field can be used for controlling the atomic transport in a ladder.

## Discussion

We have experimentally realized a generation of synthetic momentum-state lattice, where the lattice can be tuned into the noninteracting regime. We have exhibited the ability to engineer a one-dimensional synthetic lattice into a two-leg ladder geometry and implemented homogeneous and inhomogeneous synthetic gauge fields in such ladder. Based on experimental measurements of atomic populations in the ladder, we have studied in detail the homogeneous gauge field-induced chiral atomic current and its dependence on the inter-leg coupling strength. We have also demonstrated the controllable atomic transport by the inhomogeneous gauge fields.

In comparison with the previous study^42^ using two 1D momentum-state lattice to realize a two-leg ladder, we have used the NNN hopping to increase the connection between synthetic lattice sites, which enables the mapping of a single 1D momentum-state lattice into a two-leg ladder. This technique allows to implement more complex lattice models and enlarges the tool box of implementing synthetic dimensions. Moreover, we study the dependence of chiral atomic current on the inter-leg coupling. Based on our developed method and introducing atomic hyperfine states as an extra synthetic dimension, it is quite promising for exploring high-dimensional topologic physics. In contrast to previous report^42^ with the untunable interaction, our synthetic lattice that can be flexibly tuned into noninteracting regime has provided a clean platform for investigating various single-article physical models. In the future, it is quite natural to take the advantage of tunable atomic interaction in our system for studying the nonlinear interaction effect on the synthetic gauge field and exploring topological physics in presence of interactions.

## Materials and methods

A sample of cold ^133^Cs atoms is achieved by using 3D Raman sideband cooling, and then is loaded into a magnetically levitated dipole trap, which consists of two orthogonal 1064-nm lasers with the 1/*e* radii of ∼300 *µ*m, as shown in Fig. 1. Another crossed dimple trap, consists of two 1064-nm lasers focused to the 1/*e* radii of ∼58 *µ*m, is overlapped with the dipole trap. Each dimple trap laser has an angle of 12^0^ with its nearby dipole trap laser. The evaporative cooling is performed by linearly reducing the magnetic field gradient *∂B/∂z* from 31.3 G/cm to 15 G/cm for tilting the trap. Then the power of dimple laser Dim1 is ramped from 75 mW to 95 mW. In the further evaporation stage, the power of dimple laser Dim2 is lowered from 75 mW to 0, and the magnetic field gradient is continuously reduced to zero. The power of dipole laser Dip2 is finally kept at 600 mW, and the dipole laser Dip1 is switched off at the end of evaporation. An almost pure BEC is obtained in a quasi-1D optical trap, and the trapping frequencies are (*ω*_*x*_*,ω*_*y*_*, ω*_*z*_) = 2π × (120, 100, 15) Hz, where *z* is defined along the finally remaining dimple laser Dim1.

The remaining dimple laser is reflected to drive a series of Bragg transitions between the discrete momentum states. The retro-reflected laser beam passes through two AOMs, whose diffraction orders are chosen for +1 and −1. The first AOM is driven by a single-frequency rf signal, while the second AOM is driven by the multi-frequency signal. As a result, we can use the two-photon Bragg transition to couple two adjacent states with the resonance Bragg frequency $\Delta \omega _n = \omega _n^{{\rm{res}}}$. We can also couple the NNN momentum states with ∆*n* = 2 using a four-photon process by controlling the multi-frequency components. The tunneling amplitudes have been regularly calibrated by using the two-site Rabi oscillations, and the errors are guaranteed to be less than 8% for all measurements. In order to extract the distribution of atoms in the two-leg ladder, all laser fields are extinguished and the atoms are allowed to expand at the zero scattering length near 17 G. The absorption image is taken after 18 ms TOF, the population of atoms in the different momentum states can be detected directly.

## Data Availability

All experimental data and any related experimental background information not mentioned in the text are available from the authors upon reasonable request.
